# Broad Cross-Reactivity of a Microcystin ELISA Confirmed Using 19 Quantitative Reference Materials

**DOI:** 10.1021/acs.analchem.5c07801

**Published:** 2026-04-20

**Authors:** Ingunn A. Samdal, Luisa Florizoone, Kjersti L. E. Løvberg, Krista M. Thomas, Christopher O. Miles

**Affiliations:** † Norwegian Veterinary Institute, P.O. Box 64, N-1431 Ås, Norway; ‡ Metrology Research Center, 6356 National Research Council Canada, Halifax, Nova Scotia B3H 3Z1, Canada

## Abstract

Microcystins (MCs) are toxins produced by cyanobacteria and are found worldwide. Exposure may be acute or chronic through contaminated drinking water, food (such as vegetables), dietary supplements, and recreational activities. Acute exposure can cause severe liver damage due to the hepatotoxicity of MCs. More than 300 variants of MCs have been reported, making analysis complex, especially since standards are available for only a few MCs. Therefore, a rapid, cost-effective method capable of detecting all MCs in one analysis is essential to protecting public health and livestock. An antibody was raised to a mixture of five MCs creating ELISA_5b_, recognizing as many MC variants as possible in a single analysis and with as equal cross-reactivity as possible, avoiding preferential recognition of any specific MC. The updated ELISA_9b_ was obtained after additional immunization rounds and the cross-reactivities of the two ELISAs were compared using reference materials. Although good cross-reactivity was observed for both ELISAs, ELISA_9b_ demonstrated better cross-reactivity toward the 19 available reference materials of MCs and nodularin-R. Results obtained with ELISA_9b_ for water and crayfish stomach samples showed excellent correlation with those obtained with ELISA_5b_ and LC–HRMS. The limit of quantitation of ELISA_9b_ for total MCs in drinking water was 0.06 μg/L—well below the WHO’s guideline of 1 μg/L for long-term exposure. This ELISA provides a promising tool for the rapid and sensitive quantification of total MCs, including metabolites and conjugates, over a diverse range of sample types.

## Graphical abstract

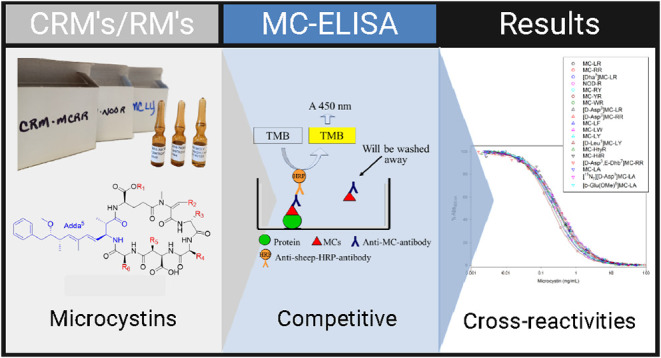

## Introduction

Microcystins (MCs) are toxins produced by many cyanobacteria worldwide. Exposure may be acute or chronic through contaminated drinking water,^1,2^ food items such as vegetables^3^ and dietary supplements,^4^ and recreational activities.^5–7^ Both human and animal health are at risk, as exposure to MCs potentially can lead to gastroenteritis, skin reactions, and liver damage.^8^ Instances of lethal poisonings have been documented across a wide array of species, including sheep, cattle, horses, pigs, dogs, fish, rodents, amphibians, waterfowl, bats, rhinoceros, wildebeest, and zebras.^9–12^ In Kenya and Tanzania, mass mortalities of Lesser Flamingos and other wildlife have been attributed to cyanotoxins.^13–15^ Although acute cyanotoxin poisoning in humans is rare due to the distinctive odor and taste of water containing high cyanobacterial concentrations, chronic exposure to low levels of cyanotoxins in drinking water may pose a significant health risk.^1,8^

MCs are cyclic heptapeptides, usually with the unusual β-amino acid 3*S*-amino-9*S*-methoxy-2*S*,6,8*S*-trimethyl-10-phenyl-4*E*,6*E*-decadienoic acid (Adda) but occasionally with a variant of Adda at position-5 (Figure 1).^16^ So far, over 300 MC analogs have been identified, with the most commonly reported MCs being MC-RR, MC-YR, MC-LR, and MC-LA.^16,17^ Nodularins (NODs) are toxic cyanobacterial cyclic pentapeptides structurally related to MCs (Figures 1 and S1), of which 10 analogs have been reported.^18^

**Figure 1:**
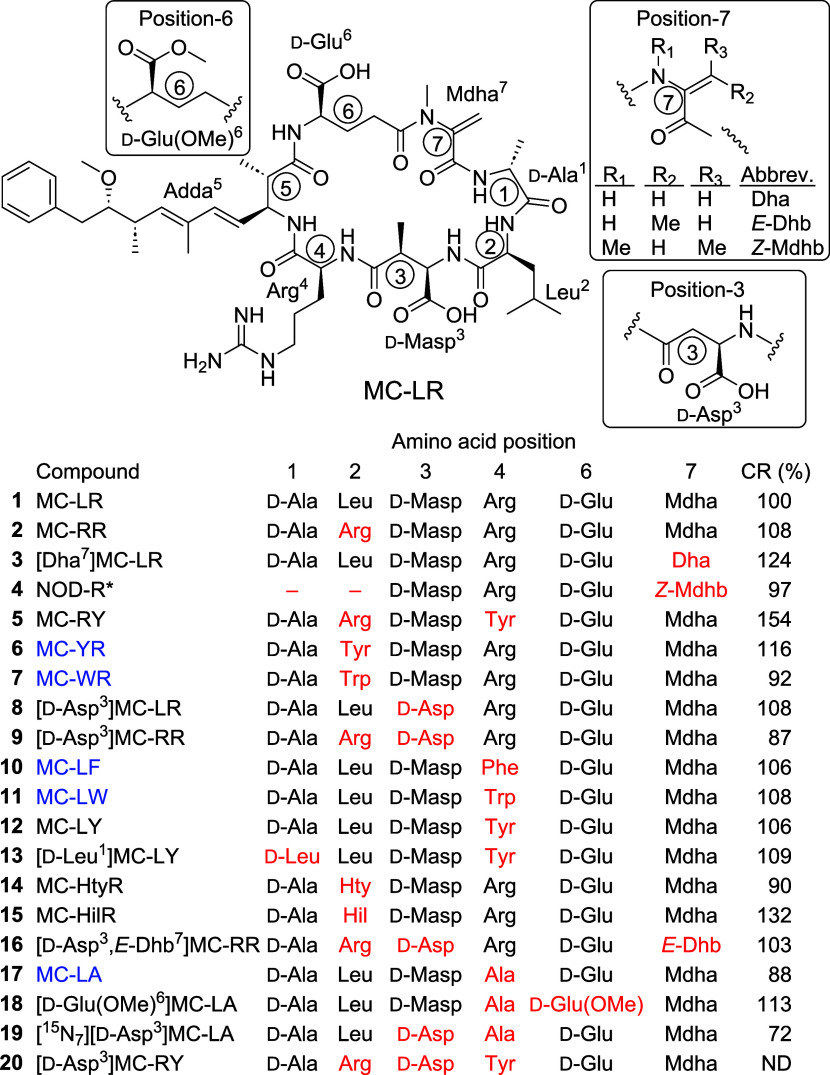
Structures of MCs and NODs included in this study, with amino acid numbering shown in circles. Structural elements that differ from those of MC-LR are indicated in red text. [d-Asp^3^]­MC-RY (**20**) was used in the plate coater, and its cross-reactivity was not tested. Microcystins used in the multihapten immunogen are shown in blue text. CR = molar cross-reactivity (see also Table 1). *Note that NOD-R contains a direct amide linkage between residues 3 and 7 and that in the standard amino acid numbering for NODs, d-Masp^3^ is denoted as amino acid-1 (Figure S1). Nomenclature follows Bouaïcha et al.^16^ and abbreviations for nonproteinogenic amino acids are listed in the “Abbreviations Used”.

The WHO recently proposed preliminary guidelines for the maximum content of 1 μg/L for total MCs in a lifetime perspective, and 12 μg/L in a short-term perspective, in drinking water, with 24 μg/L recommended in recreational waters.^1,19^ In December 2020, the EU’s drinking water directive^20^ included a maximum limit value only for MC-LR of 1 μg/L, despite many other MCs possessing similar toxicity.^16,21^ Since MCs usually occur in mixtures, and oral toxicity data for many congeners is absent or of questionable precision, it has been recommended that the maximum limit values be applied to total MCs based on the assumption that all MCs have similar toxicity to that of MC-LR.^1^

The wide array of MCs, coupled with the scarcity of standards, presents significant challenges in conducting analyses. Existing methods include high-performance liquid chromatography-ultraviolet (HPLC–UV), liquid chromatography–mass spectrometry (LC–MS), enzyme-linked immunosorbent assays (ELISAs), and bioassays. While LC–MS techniques can identify individual MC congeners, they require sophisticated instrumentation and skilled operators due to the large number of potential MCs with diverse molecular masses/transitions, require accurate standards for valid quantitation, and may not include unknown congeners. The number of MCs and NODs and the potential for these to be present in an unlimited number of combinations makes targeted LC–MS analysis extra challenging. Until recently, most of the available MC standards have been of varying quality, both qualitatively and quantitatively,^22–24^ with the notable exception of the five certified reference materials (CRMs) for MCs and NOD-R available from the National Research Council of Canada.^25–29^ However, with the repertoire of cyanotoxin CRMs increasing, along with the production of a wide range of well-characterized quantitative in-house reference materials (RMs) for MCs,^30^ it is now possible to perform reliable quantitative analyses and characterize an assay for a much wider range of MC variants.

Immunoassays utilizing appropriate antibodies hold the potential to quantify total MCs and NODs, making them ideal for screening purposes due to their high sensitivity, robustness, affordability, and ability to handle large sample numbers. Moreover, they do not necessarily require all MCs to be available as standards, provided that the assay’s cross-reactivity remains consistent across all analogs. Several ELISAs for MCs are commercially available, but data on their cross-reactivities are, unfortunately, very limited. Where data is available, significant variations in cross-reactivity among analogs have been reported,^31–33^ and quantitative reference materials are rarely utilized in such studies. Furthermore, due to the aforementioned variable quality of the available MC and NOD standards, the reported cross-reactivity data may not necessarily be very reliable. An additional problem is that MC analogs that can be present as impurities (e.g., ref 22 and we have detected “linearized” *seco*-MCs in a commercial standard of [d-Asp^3^]­MC-RR (C.O. Miles, unpublished observations)), and other impurities such as polymeric forms of MC-RY in the MC-RY reference material (Figure S3) may well cause an additional response in an immunoassay with broad cross-reactivity, even though their presence might not be detected by routine LC–MS analysis.

Most MC antibodies and ELISAs have been developed by raising antibodies against MC-LR,^32–45^ except for the initial antibody produced using MC-LA^46^ and that produced from MC-RR.^47^ This approach may result in a bias toward MCs most closely resembling the hapten, leading to inadequate recognition of some MCs.^48^ Antibodies have also been generated against the unusual β-amino acid Adda^49^ present in most MCs and NODs, including the low-toxicity ring-opened “linearized” *seco*-MCs produced during biosynthesis or hydrolysis of MCs.^16^ There is also a recent paper reporting monoclonal antibodies raised using the multihapten approach with glutaraldehyde to link the MCs to keyhole limpet hemocyanin.^50^

Our approach to generating antibodies with broad specificity toward MCs involved immunizing sheep with a carefully selected mixture of MC variants conjugated via position-7 to cationized bovine serum albumin.^51^ This strategy was adopted to avoid the recognition of specific amino acids in the most variable positions in the MC structure. The resulting polyclonal antisera were used with together with a [d-Asp^3^]MC-RY-based plate-coating antigen ([d-Asp^3^]­MC-RY (**20**)), which does not share amino acids 2, 3, or 4 with any of the immunizing haptens (Figure 1). This combination in an indirect competitive ELISA, showed exceptional sensitivity and broad specificity, performing well with cultures as well as with spiked and natural samples. The in-house protocol for preparing polyclonal antibodies is to immunize sheep 4–5 times, test bleeds for the immune response and, when the response is optimal, collect serum from a bulk bleed of ≤450 mL. Sheep are then rested for 1–2 periods to mature the antibody response, and sheep are reimmunized 1–3 times before additional bulk bleeds (≤450 mL), which generally gives antibodies with improved sensitivity/specificity. In this case, the antibodies already gave great sensitivity and specificity in ELISA_5b_ after five immunizations,^51^ resulting in sets of sera remaining untested until this work.

With more than 300 MCs and 10 NODs reported in fresh and brackish water worldwide and limited reports on cross-reactivity of these in ELISAs, we set out to test the cross-reactivity utilizing the newest antiserum (ELISA_9b_) with as many as 19 certified and in-house MC reference materials. This is the first time an ELISA for MCs has been tested for cross-reactivity with such a wide range of MCs/NODs. The performance of this new ELISA_9b_, obtained after nine immunizations, was compared to that of the originally reported ELISA_5b_,^51^ which was based on the serum obtained after five immunizations. The utility of the new ELISA_9b_ was then assessed by comparing results obtained from a natural bloom in Lake Akersvannet, Norway, with results obtained with the original ELISA_5b_, and by comparing results obtained from extracts from stomach tissues of crayfish from Lake Steinsfjorden, Norway, with results obtained by LC–HRMS analysis.

## Materials and Methods

### Materials

MC-LR utilized for ELISA optimization steps was from Enzo Life Sciences Inc. (Farmingdale, NY, USA). Certified reference materials (CRMs) of MC-LR, MC-RR, [Dha^7^]­MC-LR, NOD-R, and MC-LA,^25–29^ from the Metrology Research Centre (National Research Council Canada, Halifax, NS, Canada) were utilized for cross-reactivity studies. In-house reference materials (RMs) of MC-RY, MC-YR, MC-W*R*, [d-Asp^3^]­MC-LR, [d-Asp^3^]­MC-RR, MC-LF, MC-LW, MC-LY, MC-HtyR, MC-HilR, [d-Asp^3^,*E*-Dhb^7^]­MC-RR, and [d-Leu^1^]­MC-LY^21^ were prepared as described by Thomas et al.^30^ Two other in-house RMs were prepared in the following manner: [^15^N_7_]­[d-Asp^3^]­MC-LA was acquired from MARBIONC (North Carolina, USA), and [d-Glu­(OMe)^6^]­MC-LA was isolated from decomposed MC-LA. A stock solution of each was prepared, and identities were confirmed using LC–HRMS. No significant impurities were detected. Quantitation of these two stock solutions was performed with ^1^H NMR^52^ using benzoic acid (NIST PS1) and potassium hydrogen pthalate (NIST SRM 84L) as the external calibrants (respectively). The stock solutions were accurately diluted in 50% methanol, then dispensed into amber ampules prepurged with argon, flame-sealed, and stored at −80 °C.

Maxisorp immunoplates (96 flat-bottomed wells) were from Nunc (Roskilde, Denmark), poly­(vinylpyrrolidone) 25 (PVP) was from Serva Electrophoresis (Heidelberg, Germany), donkey-antisheep IgG (H + L)–horseradish peroxidase conjugate (antisheep–HRP) was from Agrisera Antibodies (Vännäs, Sweden), and the HRP-substrate K-blue aqueous was from Neogen (Lexington, KY, USA). The stop solution was 10% H_2_SO_4_. Inorganic chemicals and organic solvents were reagent grade or better. Plate-coating buffer was carbonate buffer (50 mM, pH 9.6). Phosphate-buffered saline (PBS) contained NaCl (137 mM), KCl (2.7 mM), Na_2_HPO_4_ (8 mM), and KH_2_PO_4_ (1.5 mM), pH 7.4. ELISA washing buffer (PBST) was 0.05% Tween 20 in PBS. Sample buffer was 10% methanol (v/v) in PBST, and antibody buffer was 1% PVP (w/v) in PBST.

### Polyclonal antisera

Polyclonal antisera were all collected from sheep 80289 at 2 weeks after each of the fifth, sixth, seventh, and ninth immunization and are referred to as sera 5b, 6b, 7b, and 9b, respectively. Immunization was done by preparing an oil-in-water emulsion of the antigen (MC-mix-cBSA, 200 μg, 1 mL) in a sterile syringe and administering this subcutaneously near the supramammary lymph node at ∼4-weekly intervals.^51^ The sheep were given two resting periods; of respectively 6 months (between the fifth and sixth immunizations), and 18 months (between the seventh and eighth immunizations).

### Plate-Coating Antigens

Chicken egg albumin (OVA) was conjugated to MCs to produce the plate-coating antigens OVA–MC-LR and OVA–[d-Asp^3^]­MC-RY as described by Samdal et al.^51^

### ELISA

All maxisorp immunoplates were coated with plate-coater antigen OVA–MC-LR or OVA–[d-Asp^3^]­MC-RY (100 μL/well) overnight in the dark at ambient temperature, washed with PBST (4 × 300 μL), blocked with 1% PVP/PBST (300 μL/well) for 1 h, and the excess was removed by washing with PBST (2 × 300 μL), as described by Samdal et al.^51^ Titers of three polyclonal antisera (6b, 7b, and 9b) were determined in noncompetitive assays on the two plate-coater antigens giving maximal absorbance of 1.0, as described by Samdal et al.,^51^ with antisheep–HRP conjugate (at 1:5500). After the substrate and stop solutions were added, absorbances were measured at 450 nm using a SpectraMax i3x plate reader (Molecular Devices, San Jose, CA, USA).

Competitive ELISAs were used to determine the optimal combination of plate-coater antigen and antiserum with MC-LR, as described by Samdal et al.^51^ Standard or sample were added in duplicate to the wells to get competition for the antibody with the plate coater. The MC-LR standard curve used was nominally 50, 16.7, 5.55, 1.85, 0.62, 0.20, 0.069, 0.023, 0.0076, and 0.0025 ng/mL in sample buffer. The optimal concentration of platecoating antigen was 0.5 μg/mL for both plate-coater antigens OVA–[d-Asp^3^]­MC-RY and OVA-MC-LR, with dilutions of antiserum 9b at 1:28000 and 1:18000, respectively.

### Cross-Reactivity

Nineteen reference materials were initially screened with the ELISA using both plate-coater antigens by preparing a dilution series of each reference material in a sample buffer. To evaluate the specificity of the optimized ELISA toward MCs and NODs, the molar *I*_50_ values (molar concentration giving 50% inhibition) were expressed as a percent *I*_50_ relative to the CRM of MC-LR.^26^ The cross-reactivity was calculated using the following formula:

$$c\mathrm{ross}‐\mathrm{reactivity}\left(\%\mathrm{CR}\right)=100\times \frac{I_{50}\mathrm{MC}‐\mathrm{LR}\left(\mathrm{CRM}\right)}{I_{50}\;\mathrm{analog}}$$

It was possible to correct for the presence of traces of other MCs in the CRMs and RMs (Tables S3 and S4) giving adjustments in cross-reactivities between 2.1 and −5.6% (mean correction of −0.5%), and these are shown in Table 1.

**Table 1:** Cross-Reactivities of the New ELISA_9b_ with a Series of Microcystin Analogs and NOD-R, Based on *I*_50_ Directly and by Considering MW^a^

compound		*n*	*I*_50_ (pg/mL)^b^	% CR	MW	*I*_50_ (nM)	molar % CR^c^	%CV	corr % CR^d^
MC-LR	CRM^26^	5	287	100	995.2	288	100	6	100
MC-RR	CRM^27^	3	282	102	1038.2	272	106	7	108
[Dha^7^]MC-LR	CRM^25^	3	233	124	981.2	237	122	6	124
NOD-R	CRM^28^	3	248	116	825.0	300	97	9	97
MC-LA	CRM^29^	3	305	94	910.1	335	86	3	88
MC-RY	RM^30^	3	184	156	1045.2	176	164	3	154
MC-YR	RM^30^	3	261	111	1045.2	249	117	13	116
MC-W*R*	RM^30^	3	327	88	1068.2	306	94	3	92
[d-Asp^3^]MC-LR	RM^30^	3	268	108	981.2	273	106	11	108
[d-Asp^3^]MC-RR	RM^30^	3	327	88	1024.2	319	90	3	87
MC-LF	RM^30^	3	255	113	986.2	259	112	8	106
MC-LW	RM^30^	3	274	107	1025.2	267	110	16	108
MC-LY	RM^30^	3	271	106	1002.2	271	107	7	106
[d-Leu^1^]MC-LY	RM^30,21^	3	282	102	1044.3	270	107	9	109
MC-HtyR	RM^30^	3	345	83	1059.2	326	89	6	90
MC-HilR	RM^30^	3	215	134	1009.2	213	136	3	132
[d-Asp^3^,*E-*Dhb^7^]MC-RR	RM^30^	3	296	99	1024.2	289	102	17	103
[d-Glu(OMe)^6^]MC-LA	RM	4	234	123	924.1	260	115	15	113
[^15^N_7_][d-Asp^3^]MC-LA	RM	3	366	79	903.8	405	71	6	72

a The LOD (*I*_20_) obtained for the CRM of MC-LR was 0.06 ng/mL in the absence of matrix effects.

b *I*_50_ values are the concentrations of analogs giving 50% inhibition of binding of antibody to the coating antigen (OVA–[d-Asp^3^]­MC-RY).

c Calculated based on *I*_50_ adjusted for MW.

d Cross-reactivity in the CRMs and in-house RMs when corrected for MC-related impurities.

### Testing with Samples

Two sets of samples were used for testing and verifying ELISA_9b_ on plate-coater OVA–[d-Asp^3^]­MC-RY. Water samples were collected from Lake Akersvannet (Vestfold, Norway) in the summer of 2022.^53^ This lake has regular presence of cyanobacteria, including *Microcystis* spp. The samples were quantified at different dilutions using sample buffer depending on the concentration of MCs present in the sample (Table S2) to get them into the assay’s working range (between *I*_20_ and *I*_80_). Dilutions were 1:5 for the water samples with the lowest levels and up to 1:20480 for the highest sample. The results with the optimized ELISA_9b_ were compared to the results using the ELISA_5b_, the performance of which has been verified for cyanobacterial cultures using LC–MS.^51^

Crayfish stomach samples from Lake Steinsfjorden in 2015 were gathered and extracted with methanol (1:9 w/v), as described by Samdal et al.,^54^ and stored sealed at −20 °C between analyses. Lake Steinsfjorden has regular blooms of *Plankthothrix* spp. The crayfish stomach samples were analyzed with the new ELISA_9b_ at a minimum of 1:100 dilution, abolishing any matrix effects as shown previously.^54^ The optimized ELISA_9b_ was compared to the LC–HRMS analysis^55^ of the same extracts from crayfish from Lake Steinsfjorden in 2015.

## Results and Discussion

A rapid and cost-effective ELISA with the recognition of all MC analogs with cross-reactivities paralleling their human toxicological potency would be ideal. But most MCs are poorly described with respect to toxicity, and the measurement uncertainty for relative toxicity is probably large, especially given the questionable quality of the standards often used for toxicological studies. Our approach that has therefore been to produce an ELISA with as equal response as possible to the various intact MC analogs. This approach is based on the precautionary principle, as research indicates that all intact MCs biosynthesized by cyanobacteria that have been tested have exhibited toxicity either in vivo or in vitro.^1,19^ The ELISA is also designed to accommodate potential future discoveries of new toxic MC analogs that, based on the past experience, will inevitably continue to be found.^16,17^ Such novel MCs are likely to be recognized by antibodies with broad specificity, such as those employed in this study, but they might not be detected using standard LC–MS/MS screening methodologies as demonstrated by Miller et al.^4^

### Matured Antibody Response and ELISA Optimization

Although the antiserum obtained after five immunizations worked very well in ELISA_5b_,^51^ the subsequent immunizations were nonetheless performed as planned, with two resting periods in between. However, the antisera from these later immunizations had not been evaluated for use in ELISAs previously.

As with the previously published ELISA,^51^ to be compatible with most standard extraction methods for cyanobacterial algal toxins, the ELISA was optimized using 10% methanol in both samples and standards. To maximize ELISA sensitivity, the concentration of reagents needed to be optimized for each serum and plate-coater. Criteria for optimization were *A*_max_, slope of the curve, *I*_50_, working range (*I*_20_–*I*_80_), and limit of quantitation (LOQ), based on *I*_20_ and multiplied by the dilution factor (i.e., 1.2 for water and culture samples and 10 for methanolic extracts).

The four antisera were tested through checkerboard titrations and optimization of standard curves on 0.5 μg/mL of the OVA–[d-Asp^3^]­MC-RY and OVA–MC-LR plate coaters using a semiquantitative MC-LR standard, resulting in the standard curves on OVA–[d-Asp^3^]­MC-RY shown in Figure 2. The standard curve for the fifth serum had a nominal *I*_50_ of 0.213 ng/mL and a limit of quantitation (*I*_20_) of 0.04 ng/mL. The standard curves from the sixth and seventh sera were almost identical, but slightly less sensitive than the original ELISA_5b_.^51^ The standard curve of ninth serum (at 1:28,000 dilution) gave the most sensitive standard curve, with a nominal *I*_50_ at 0.158 ng/mL and *I*_20_ at 0.05 ng/mL. As a semiquantitative standard was used, values are correct relative to each other but are not necessarily correct in absolute terms. Therefore, valid assay performance characteristics needed to be established with quantitative RMs and CRMs.

**Figure 2:**
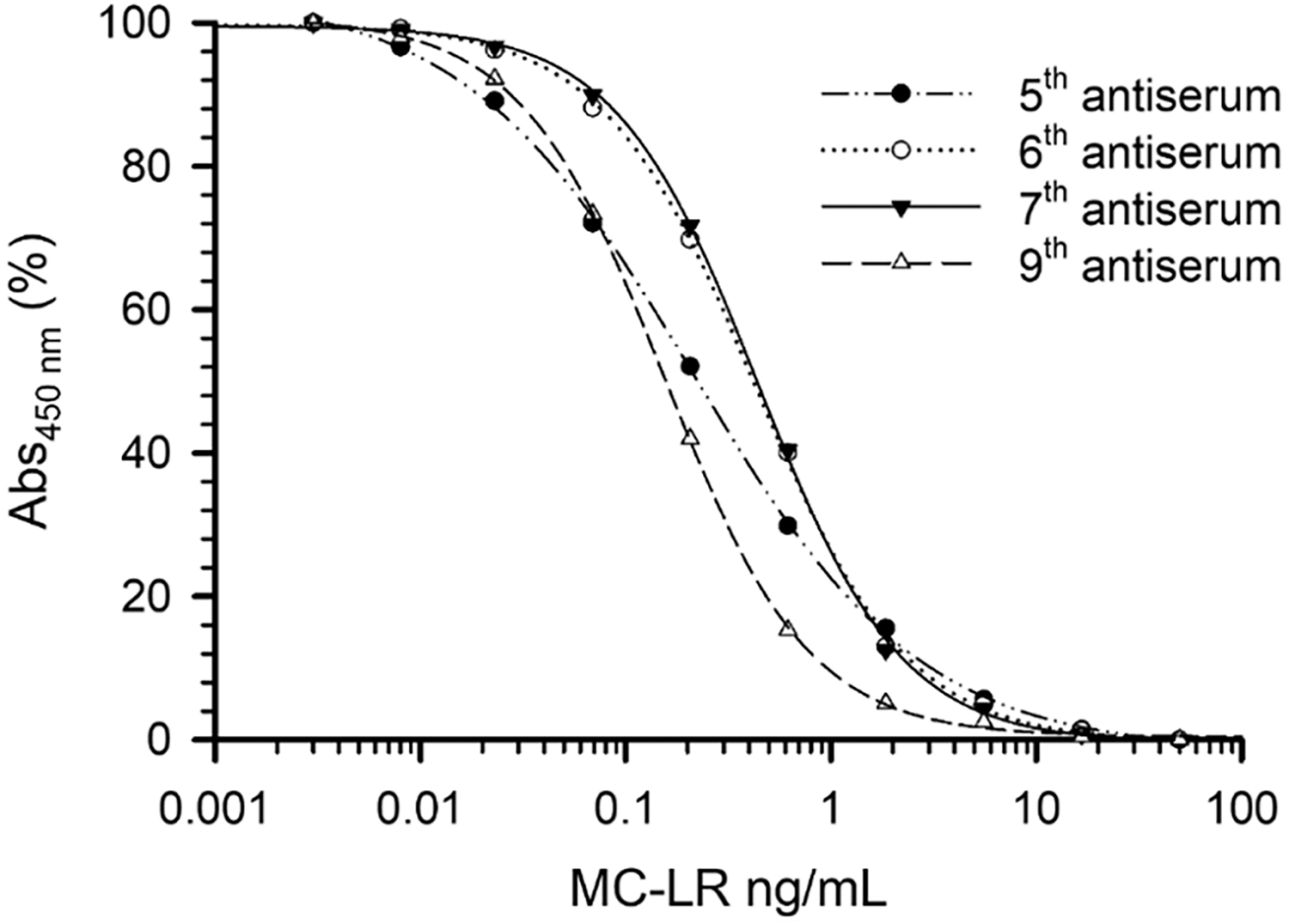
MC-LR standard curves in optimized ELISAs using four antisera from sheep 80289 (after the 5th, 6th, 7th, and 9th immunizations) on plate-coating antigen OVA–[d-Asp^3^]­MC-RY. The *I*_20_ values were for the 5th serum at 0.04 ng/mL, for the 6th serum at 0.13 ng/mL, for the 7th serum at 0.14 ng/mL, and for the 9th serum at 0.05 ng/mL. *I*_20_ is the limit of quantification without considering matrix effects. The curves were obtained with a semiquantitative standard of MC-LR and are therefore indicative rather than quantitative.

### Antibody Specificity

A multihapten approach was chosen in order to obtain antisera with broad, but nonetheless specific, recognition of MCs regardless of the amino acids present in positions 2, 3, and 4, as previously described for the ELISA developed with the fifth antiserum.^51^ To evaluate the success of this strategy, the cross-reactivity of available quantitative MC and NOD standards was tested with the CRM of the MC-LR standard curve set to 100% cross-reactivity on both plate coaters. Standard curves of MC-RR, [Dha^7^]­MC-LR, NOD-R, and MC-LA (all CRMs), and MC-RY, MC-YR, MC-W*R*, [d-Asp^3^]MC-LR, [d-Asp^3^]MC-RR, MC-LF, MC-LW, MC-LY, MC-HtyR, MC-HilR, [d-Asp^3^,*E*-Dhb^7^]­MC-RR, [d-Glu­(OMe)^6^]­MC-LA, [d-Leu^1^]MC-LY, and [^15^N_7_]­[d-Asp^3^]­MC-LA (all in-house RMs) were obtained as for the CRM of the MC-LR. All MCs and NOD-R showed concentration-dependent inhibition of antibody binding in the ELISA_9b_ on both plate coaters. As the most group-specific plate-coater antigen was found to be OVA–[d-Asp^3^]MC-RY based on initial screening with the CRMs and in-house RMs (Table S1), the full cross-reactivity screening was done on plate coater OVA–[d-Asp^3^]­MC-RY, and the resulting inhibition curves are shown in Figure 3. The array of MCs and NOD-R used in the cross-reactivity testing included variants of all but one (Adda^5^) of the seven amino acids present in the MC skeleton (Figure 1).

**Figure 3:**
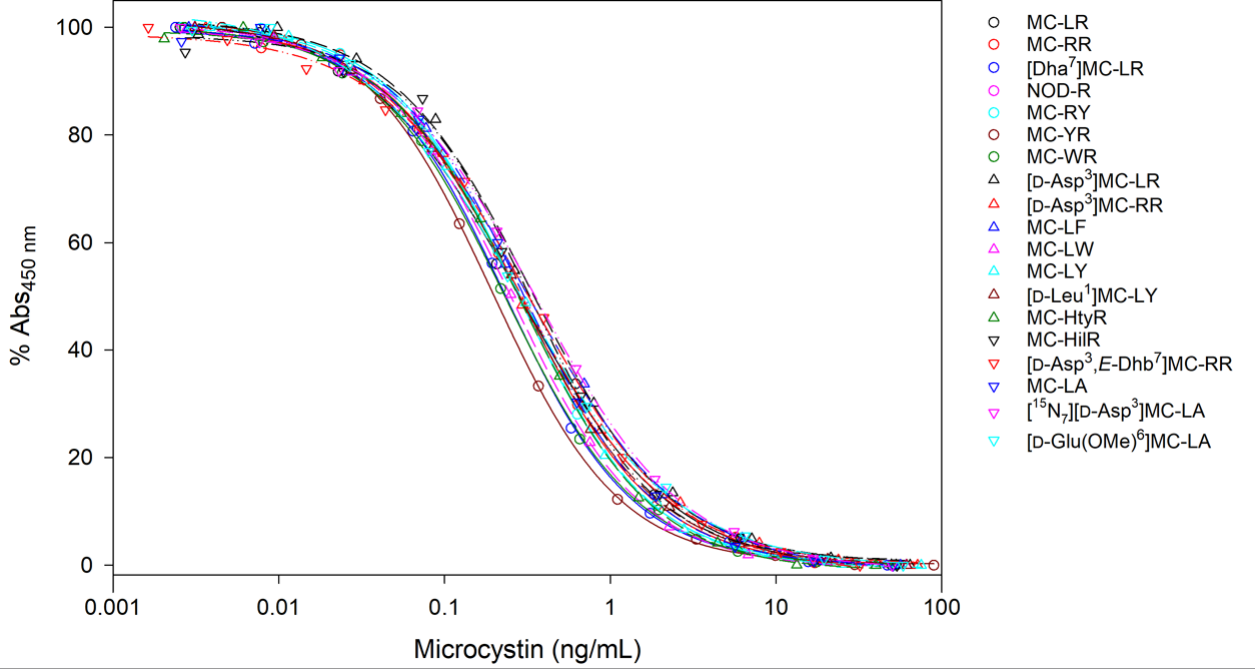
ELISA inhibition curves with 19 MCs and NOD-R CRMs^25–29^ and RMs^30^ using antiserum 9b with OVA–[d-Asp^3^]­MC-RY as plate coater.

Cross-reactivities were calculated based on molar *I*_50_ values from 3 curves for each MC-standard compared to the mean of *I*_50_ values from 5 curves obtained from CRM of the MC-LR. The molar cross-reactivities relative to MC-LR varied between 71 and 164% (Figure 4), with a median of 106% for all RMs/CRMs of MCs and NOD-R (Table 1). Among these, the cross-reactivities of MC-RR, MC-YR, MC-LA, and NOD-R were 106%, 117%, 86%, and 97%, respectively. The highest cross-reactivities were obtained for MC-RY (164%) and MC-HilR (136%), while the lowest was for [^15^N_7_]­[d-Asp^3^]­MC-LA (71%). Most of the other MCs had cross-reactivities of 80–120% with at least one of the three obtained curves. The originally published ELISA_5b_ had cross-reactivities of 41–100% (median 66%), with MC-RR, MC-YR, MC-LA, and NOD-R having cross-reactivities of 80%, 41%, 42%, and 72%, respectively,^51^ although some of these cross-reactivities were not obtained from quantitative RMs. Thus, the optimized ELISA_9b_ had excellent cross-reactivity with all MCs tested. The apparently high cross-reactivity toward MC-RY is likely attributable to the presence of significant amounts of dimers and trimers of MC-RY (**5**) (Figure S3), as reported elsewhere.^16^ Only the concentration of the monomeric form was estimated with the quantitation approach used for producing the in-house RMs,^30^ whereas the antibodies would be expected to bind to both the polymeric and monomeric forms. There are trace impurities in the five CRMs (Table S3) that are expected to influence the cross-reactivity; however, including a correction for these changes the calculated cross-reactivities of the MCs by only 0–2.4%. Impurities in some of the in-house RMs were found to be higher (Table S4). Including a correction for these adjusts the cross-reactivities down by 1–8%. Overall, the CRMs and RMs would then be adjusted between 2.1 and −5.6% with a mean correction of −0.5% (Table 1). This adjustment is not considered significant as normal variability inbetween ELISA plates is typically considered to be up to 15–20% and still acceptable (Figure 4).^56,57^

**Figure 4:**
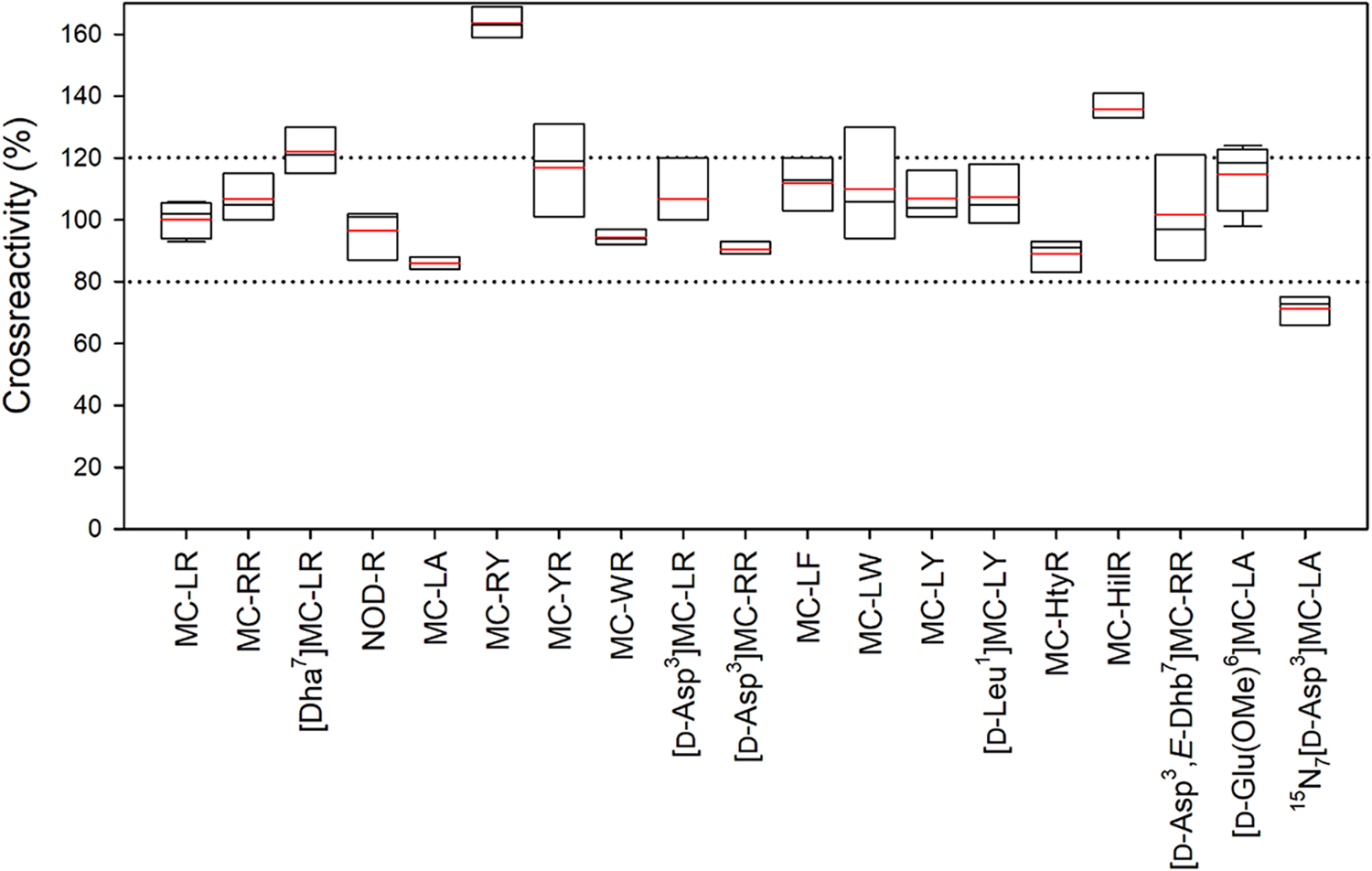
Boxplot of the molar cross-reactivities (%) of ELISA_9b_ toward quantitative standards of 19 MCs/NOD-R. The dark lines are the median values, the boxes indicate maximum and minimum values, and the red lines are the mean values (*n* = 3, except for MC-LR, where *n* = 5, and [d-Glu­(OMe)^6^]­MC-LA where *n* = 4). The dotted lines show 80% and 120% cross-reactivities.

### Analysis of Naturally Contaminated Samples

#### Lake Water Samples

To verify its suitability for analyzing water samples, a set of 22 water samples collected from Lake Akersvannet in summer 2022 ^53^ were analyzed using both antiserum 5b^51^ and antiserum 9b on plate coater OVA–[d-Asp^3^]MC-RY (Figure 5). The ELISA_9b_ results were about 1.2-fold higher than those obtained with ELISA_5b_. A high level of correlation (*y* = 1.11 *x*, *R* ^2^ = 0.997) was observed between the two methods. When excluding the two highest samples with respect to MCs, a small decrease in correlation (*y* = 1.47 *x*, *R* ^2^ = 0.885) was obtained (Figure 5B). The slightly higher MC concentrations obtained when using antiserum 9b are consistent with its broad cross-reactivity toward MCs, as indicated in Table S2.

**Figure 5:**
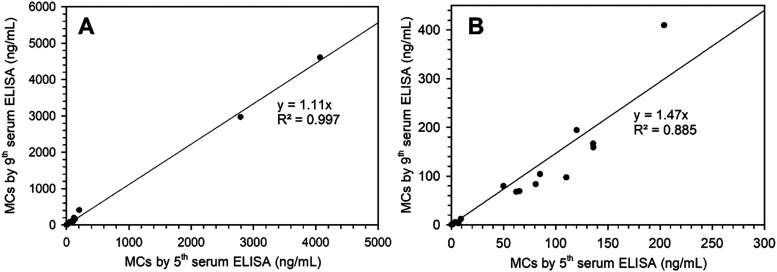
Comparison of total MCs in water samples from Lake Akersvannet from summer 2022 as determined by ELISA_5b_ vs the new ELISA_9b_. (A) All 22 samples; (B) the lowest 20 of the 22 samples.

#### Crayfish Samples

To confirm its applicability to real samples with a more challenging matrix and complex MC-profiles, 15 European Noble Crayfish stomach samples from Lake Steinsfjorden during and after a bloom of *Planktothrix* spp. in summer–autumn 2015 ^54^ were analyzed by both LC–HRMS^55^ and ELISA_5b_.^54^ When we compared the ELISA_9b_ results with the LC–HRMS results for the sum of only the four most commonly reported MCs (MC-RR, MC-LR, MC-YR, and MC-LA) (Figure 6A), the correlation was poor (*y* = 2.84 *x*; *R* ^2^ = 0.835). The correlation improved (*y* = 2.18 *x*; *R* ^2^ = 0.863) when all MCs known prior to the crayfish study were included in the LC–HRMS analysis^55^ (Figure 6B). However, when all MCs detected in the LC–HRMS analysis were included in the comparison, including the novel variants MC-LHnv, MC-LNao, and MC-LCit^55^ (suspected to be crayfish metabolites of known MCs), the correlation between the methods was excellent (*y* = 1.109 *x*; *R* ^2^ = 0.995) (Figure 6C). This shows that what are often considered as ELISA matrix effects in samples, or “overestimations” by ELISA methods, may sometimes be due to real MCs not detected by targeted LC–MS methods. Indeed, Miller et al.^4^ recently identified the unanticipated presence of MC-HtyR in a cyanobacterial food supplement due to initial discrepancies in the quantitation of MCs by LC–MS/MS, PPI assay, and ELISA_5b_. However, the correlation between the methods was very good when MC-HtyR was subsequently included in the LC–MS/MS analysis.^4^

**Figure 6:**
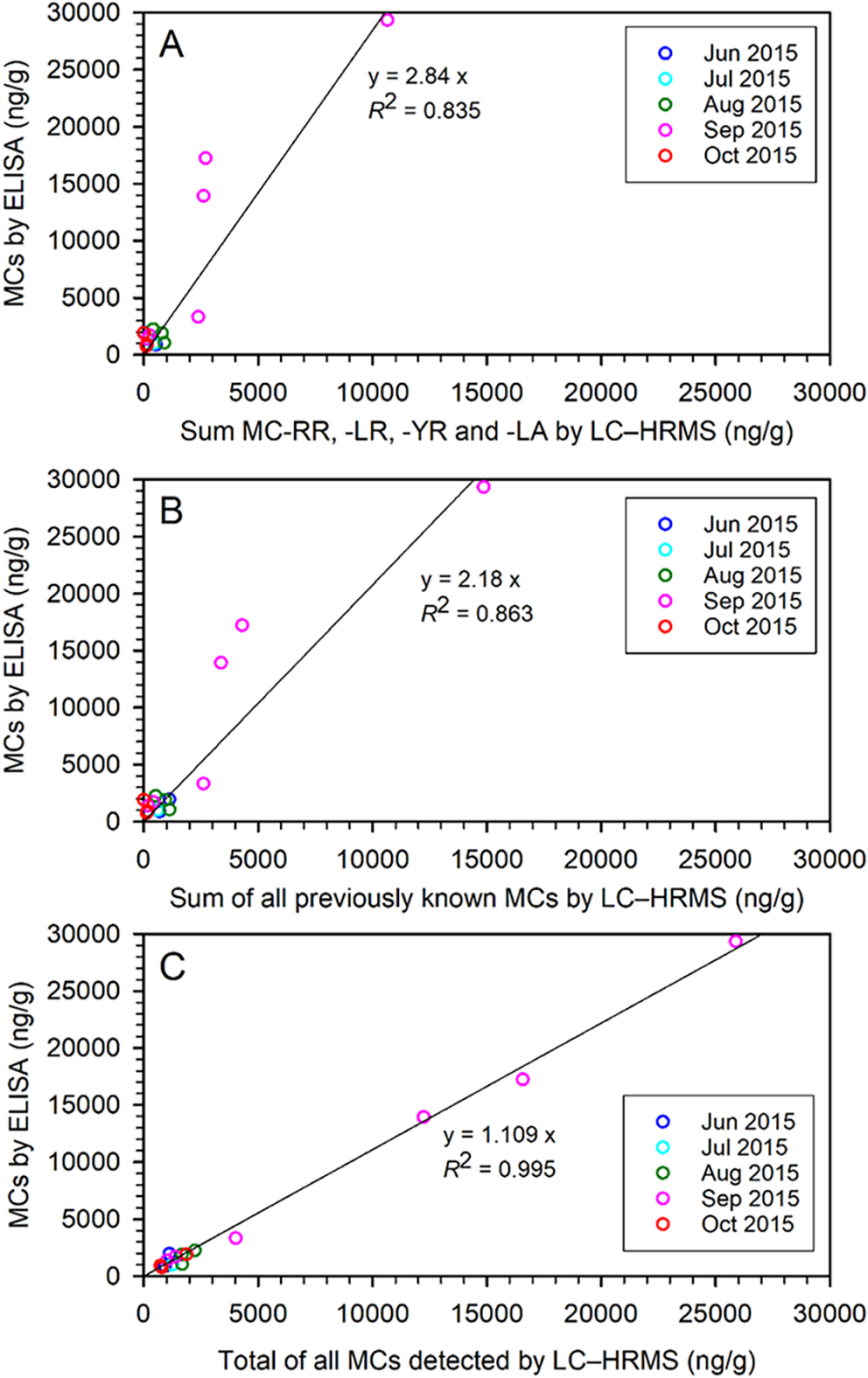
Comparison of total MCs determined by ELISA_9b_ (this study) and LC–HRMS^55^ in extracts from the stomachs of 15 wild-caught European Noble Crayfish *Astacus astacus* from Lake Steinsfjorden in Norway in Jun–Oct 2015. Concentrations of MCs from ELISA_9b_ are plotted against the: (A) sum of MC-RR, MC-LR, MC-YR, and MC-LA by LC–HRMS; (B) sum of known MCs^16^ prior to the crayfish analysis by LC–HRMS; and (C) total MC concentrations detected by LC–HRMS (LC–HRMS data from ref 55). The lines shown are linear least-squares fits to the data, along with the equation for the fitted line and its *R* ^2^ value.

The correlation between ELISA_9b_ and the sum of all MCs detected by LC–HRMS was very good for all months of the study, even for crayfish samples containing lower concentrations of MCs (Figure 7). This also confirms the universally good cross-reactivity with the antibodies, since the MC-profile in the crayfish changed markedly from June to October^55^ while the correlation between the results of the new ELISA method and the LC–HRMS analysis remained essentially constant (Figure 6C).

**Figure 7:**
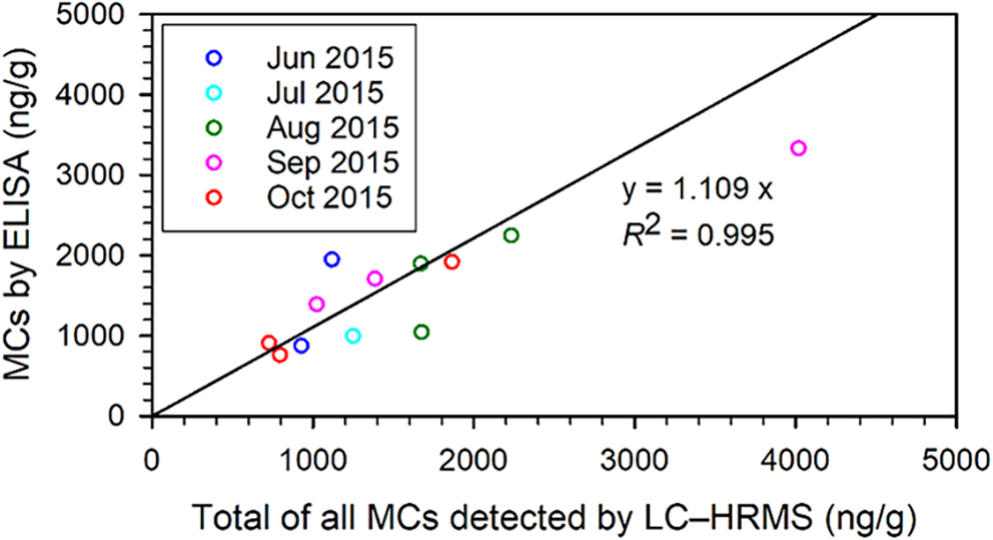
Comparison of total MCs determined by ELISA_9b_ (this study) and by LC–HRMS^55^ in extracts of stomachs of 15 wild-caught European Noble Crayfish (*A. astacus*) from Lake Steinsfjorden, Norway, for the 12 samples from Figure 6C with MC concentrations below 5000 ng/g. The fitted line is from the full data set, as shown in Figure 6C.

Given the excellent cross-reactivity characteristics demonstrated here for ELISA_9b_ (Figures 3 and 4, Table 1), the very good correlation observed between total MCs by ELISA and LC–HRMS in crayfish stomachs (Figures 6C and 7) also supports the approach used for estimating the concentrations of the 38 MCs identified by LC–HRMS in the crayfish.^55^ For most of these MCs, no standards of any kind were available, so the authors used three representative quantitative CRMs containing two, one, or no Arg groups (MC-RR, MC-LR, and [d-Leu^1^]­MC-LY), respectively, and full-scan LC–HRMS analysis in positive ionization mode to estimate concentrations of MCs with the corresponding Arg content. The good correlation obtained suggests that this approach could provide a practical and acceptable approximation of the total MC content provided that all congeners are identified and included in the LC–HRMS analysis.

#### Quantitative Reference Materials

This study highlights the importance of reliable quantitative standards, not only for instrumental analyses, such as LC–MS, but also for the evaluations of toxicities and assay performance. Very few toxicity evaluations (e.g., bioassays and protein phosphatase inhibition (PPI) assays) have been performed with calibration against genuinely quantitative standards. Given the documented variable quality of most commercially available MC standards,^22–24^ many of the resulting relative toxicities and EC_50_ values must be considered only semiquantitative. The same considerations also apply to LC–MS and immunoassay cross-reactivity studies based on such standards.

The CRMs used here were assigned accurate concentrations using one or more primary methods,^25–29^ are metrologically traceable, and have assigned uncertainties. The in-house RMs used were assigned concentrations using a single primary quantitative method.^30^ For the purposes of this study, the concentrations of all of the CRMs and RMs were corrected for any impurities that were detected by full-scan LC–HRMS.

Instrumental analytical methods detect individual toxin variants, providing information about the toxin profile that is not available from immunoassays or PPI assays. While this can be very useful, it also means that if multiple analogs are present, some of these may be unidentified or below the limit of quantitation of the instrumental analytical method. In contrast, immunoassays and PPI assays provide an indication of the total concentration of MCs and NODs, but there is no information as to which variants are present. Therefore, instrumental analytical methods, immunoassays, and PPI assays complement each other by giving different (but correct) information about MC content in samples. Good examples of this are the finding of MC-HtyR in supplements^4^ and the novel variants MC-LHnv, MC-LNao, and MC-LCit reported in crayfish.^55^ In both cases, the correlation between the methods was very good when these new MCs were included in the LC–MS analysis.

## Conclusion

The optimized ELISA_9b_ is a rapid, sensitive, and low-cost method for routine detection of MCs/NODs in water, crayfish tissues, and most likely other types of samples as well. The ELISA_9b_ detects all 19 tested variants and shows broad MC-specific cross-reactivity (72–154%, median 106%), a significant improvement on the original ELISA_5b_. The ELISA's strong correlation with LC–HRMS confirms good cross-reactivity to the sometimes dominant novel MC variants found by LC–HRMS in European Noble Crayfish from Lake Steinsfjorden.

With an assay sensitivity at 0.06 ng/mL, most samples must be diluted into the assay’s working range, typically 5–10-fold for lake water samples and 100-fold for crayfish extracts.

Given the high sensitivity and specificity for MCs/NODs, indirect competitive ELISA_9b_ has the potential to be a great tool for studying naturally contaminated samples, complementary to LC–MS analysis. Although based on a single-batch of polyclonal antiserum, one 450 mL sheep-blood collection yields enough antiserum for about one million ELISA plates, ensuring long-term usability.

## Supplementary Material



## Notes

Structures of NODs (Figure S1); boxplot of the cross-reactivities toward 18 MC standards and NOD-R standard based on *I*_50_ nM (Figure S2); LC–HRMS chromatogram and spectra of standard containing MC-RY (Figure S3); molar cross-reactivity of the ELISA with two plate coaters (Table S1); and samples collected in Lake Akersvannet in 2022 (Table S2); information on minor impurities in the MC and NOD CRMs (Table S3); information on the MC RMs and an estimate of minor impurities (Table S4) (PDF)

## Abbreviations

Adda: 3*S*-amino-9*S*-methoxy-2*S*,6,8*S*-trimethyl-10-phenyl-4*E*,6*E*-decadienoic acid
CRM: certified reference material
Dha: dehydroalanine
Dhb: dehydrobutyrine
ELISA: enzyme-linked immunosorbent assay
Glu­(OMe): glutamic acid 1-methyl ester
HRP: horseradish peroxidase
HPLC–UV: high-performance liquid chromatography–ultraviolet detection
Hil: homoisoleucine
Hty: homotyrosine
*I*_20_: 20% inihibition of the ELISA assay
*I*_80_: 80% inihibition of the ELISA assay
LC–HRMS: liquid chromatography–high-resolution mass spectrometry
LC–MS: liquid chromatography–mass spectrometry
Masp: *erythro-*β*-*methylisoaspartate
MC: microcystin
Mdha: *N*-methyldehydroalanine
Mdhb: *N*-methyldehydrobutyrine
NOD: nodularin
OVA: chicken egg albumin
PBS: phosphate-buffered saline
PBST: PBS with 0.05% Tween 20
PPI: protein phosphatase inhibition
PVP: polyvinylpyrrolidone 25
RM: reference material
