# Supplementary material for: Broad Cross-Reactivity of a Microcystin ELISA Confirmed Using 19 Quantitative Reference Materials
Source: Anal Chem. 2026 Apr 20;98(17):12466–75. doi: 10.1021/acs.analchem.5c07801 (PMC13150811; doi:10.1021/acs.analchem.5c07801)
Supplement: Supplementary file 1 [file ac5c07801_si_001.pdf]

Supporting information

Broad cross-reactivity of a microcystin ELISA confirmed using nineteen quantitative reference materials

Ingunn A. Samdal[1,*], Luisa Florizoone[1], Kjersti L. E. Løvberg[1], Krista M. Thomas[2], and Christopher O. Miles[1,2]

[1]Norwegian Veterinary Institute, P.O. Box 64, N-1431 Ås, Norway
[2]Metrology Research Center, National Research Council Canada, Halifax, NS, B3H 3Z1, Canada

Table of Contents

Figure S1	Structures of NODs	S2
Figure S2	Boxplot of the cross-reactivities toward 18 MC-standards and a NOD-R based on I_{50} nM	S3
Figure S3	LC–HRMS chromatogram and spectra of standard containing MC-RY	S4
Table S1	Molar cross-reactivity of the ELISA with two plate coaters	S5
Table S2	Samples collected in Lake Akersvannet in 2022	S6
Table S3	Information on minor impurities in the MC and NOD CRMs	S7
Table S4	Information on the MC RMs and an estimate of minor impurities	S8

Supporting information

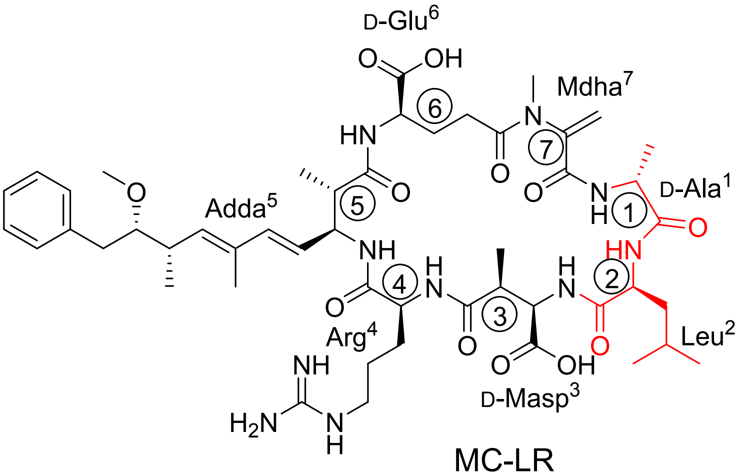

MC-LR

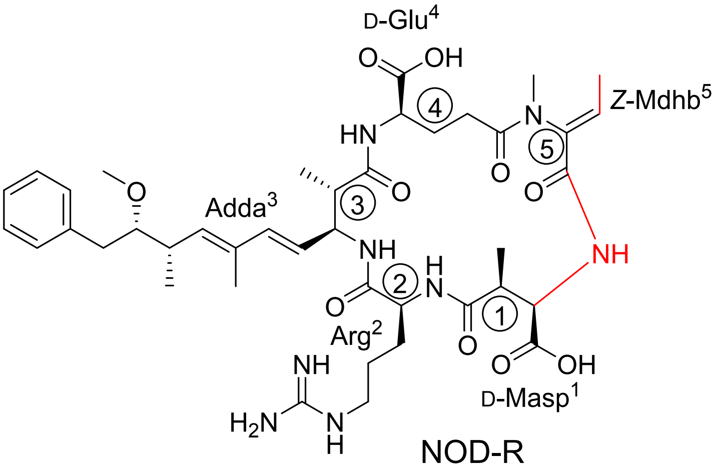

NOD-R

Figure S1. Structure of NOD-R (bottom) used in this study, with amino acid numbering shown in circles. The structure of MC-LR is shown above it, for comparison. Structural elements that differ between the two cyanotoxins (D-Ala and Leu are absent, and an additional methyl group is present on the olefinic methylene to form *Z*-Mdhb, in NOD-R) are coloured red. Note the different numbering conventions for NODs and MCs such that, for example, NOD-R contains an Adda³-residue, whereas MC-LR contains an Adda⁵-residue, despite their structural similarity.

Supporting information

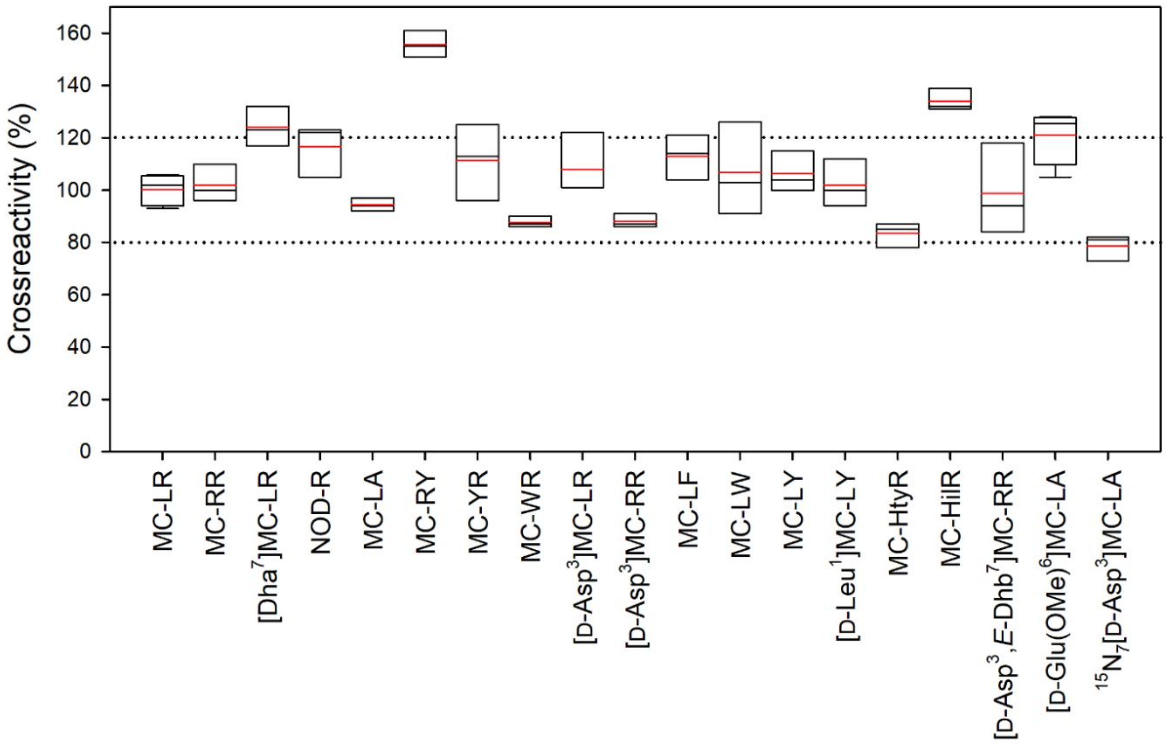

Figure S2. Boxplot of the cross-reactivities (%) toward 18 MC-standards and a NOD-R standard from NRC in MC-ELISA$_{9b}$ based on I_{50} without considering their molecular weight. The dark lines are the median values, the boxes indicate maximum and minimum values, and the red lines are the mean values (n = 3, except for MC-LR where n = 5 and [D-Glu(OMe)^6] MC-LA where n = 4). Cross-reactivity = 100 × (I_{50} CRM-MC-LR)/(I_{50} analogue). The dotted lines show 80% and 120% cross-reactivities.

Supporting information

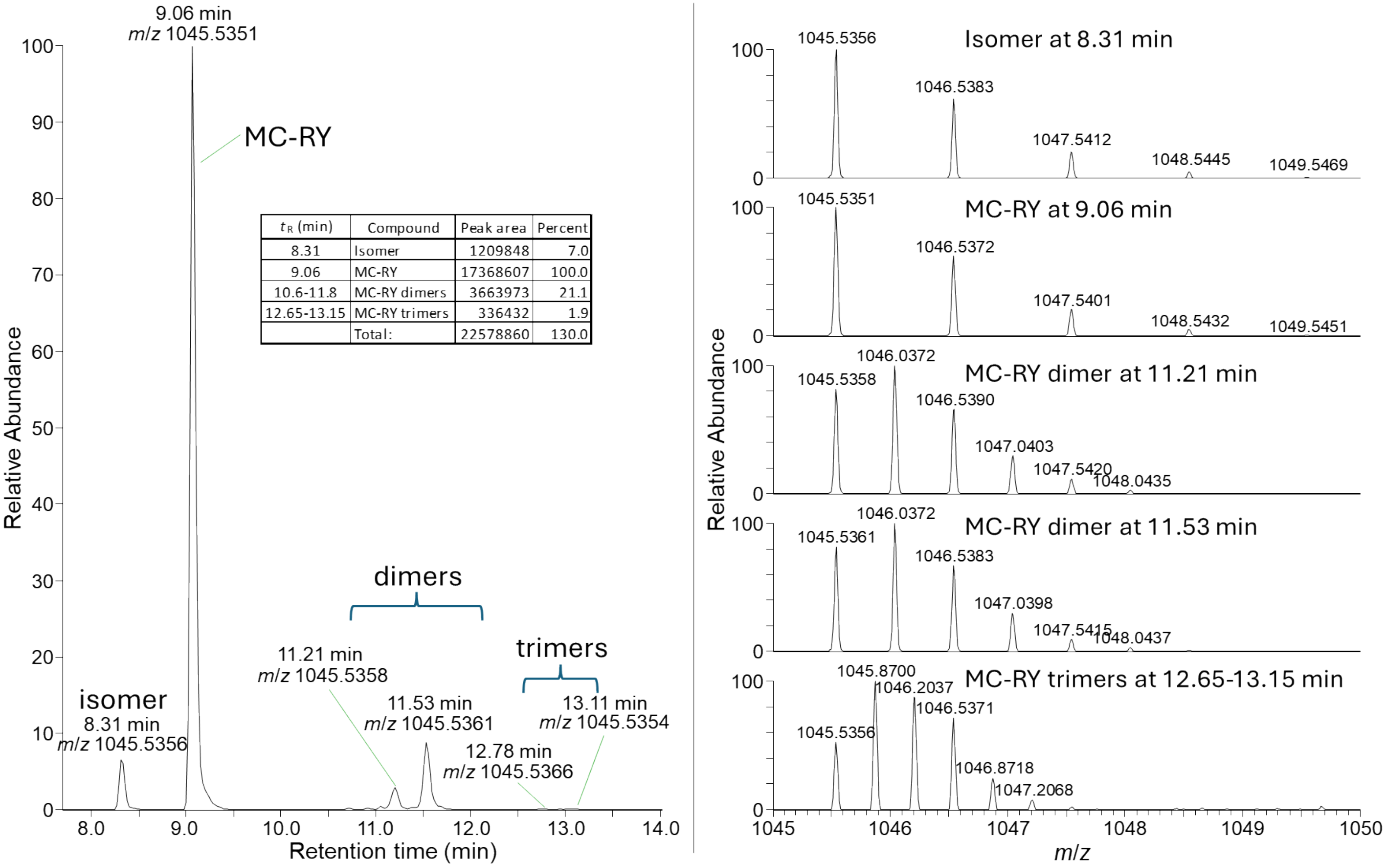

t_R (min)	Compound	Peak area	Percent
8.31	Isomer	1209848	7.0
9.06	MC-RY	17368607	100.0
10.6-11.8	MC-RY dimers	3663973	21.1
12.65-13.15	MC-RY trimers	336432	1.9
	Total:	22578860	130.0

Figure S3. Left, extracted-ion (*m/z* 1045.5–1048.5) full-scan LC–HRMS chromatogram of a standard containing RM-MCRY (and no MC-YR) in positive ionization mode under conditions described by Miles et al.[1] Areas of the various peaks are shown in the inset along with their abundance relative to MC-RY. To the right are shown mass spectra of selected peaks in the chromatogram

Supporting information

Table S1. Molar cross-reactivity of the $ELISA_{9b}$ (using antiserum 80289-9b), on plate coater antigens OVA–MC-LR and OVA−[D-Asp[3]]MC-RY, with a series of microcystin analogues.

MC analogues	MW	OVA-MC-LR			OVA−[D-Asp[3]]MC-RY		
		n[a]	IC_{50} (nM)	CR (%)	n[a]	IC_{50}[b] (nM)	CR[c] (%)
MC-LR[CRM]	995.2	3	0.275	100	5	0.228	100
MC-RR[CRM]	1038.2	1	0.328	84	3	0.175	106
[Dha[7]]MC-LR[CRM]	981.2	1	0.248	111	3	0.154	122
NODR[CRM]	825.0	1	0.312	88	3	0.190	97
MC-LA[CRM]	910.1	/	/	/	3	0.116	86
MC-RY	1045.2	1	0.191	144	3	0.154	164
MC-YR	1045.2	1	0.297	93	3	0.204	117
MC-WR	1068.2	1	0.362	76	3	0.193	94
[D-Asp[3]]MC-LR	981.2	1	0.285	96	3	0.216	106
[D-Asp[3]]MC-RR	1024.2	1	0.378	73	3	0.165	90
MC-LF	986.2	1	0.312	88	3	0.176	112
MC-LW	1025.2	1	0.340	81	3	0.175	110
MC-LY	1002.2	1	0.320	86	3	0.179	107
[D-Leu[1]]MC-LY	1044.3	1	0.348	79	3	0.222	107
MC-HtyR	1059.2	1	0.445	62	4	0.141	89
MC-HilR	1009.2	1	0.201	137	3	0.210	136
[D-Asp[3],*E*-Dhb[7]]MC-RR	1024.2	1	0.277	99	3	0.224	102
[D-Glu(OMe)[6]]MC-LA	924.1	/	/	/	3	0.193	115
[$^{15}N_7$][D-Asp[3]]MC-LA	903.8	/	/	/	3	0.260	71

[a]Number of individual screenings; [b]Average I_{50} values; [c]Average CR.

Supporting information

Table S2. Samples collected in Lake Akersvannet in 2022. “n” refers to number of dilutions in ELISA used to determine concentration of MCs in the sample. Dilutions are the dilution of the original samples that were within the ELISA standard curve and thereby valid for calculation.

Samples	5th bleed MCs (ng/mL)	9th bleed MCs (ng/mL)	n	Dilutions
NA5	9.27	12.2	2	5, 10
NL6	0.84	0.97	2	10, 20
SVA7	3.51	3.88	2	10, 20
SVA8	0.93	1.47	3	5, 10, 20
NA8	1.33	1.59	3	5, 10, 20
NA9	0.27	0.27	1	5
SØA10	3.89	5.42	1	20
SOA15	6.97	3.12	2	10, 20
SØA16	1.43	1.05	2	5, 10
NA7	1.35	1.07	2	5, 10
VA5	85.09	104.1	1	320
SVA6	136.0	159.2	1	320
SØA8	50.0	79.6	1	320
SVA9	80.9	83.6	2	160, 320
SØA9	65.2	69.0	2	160, 320
SØA12	120.2	194.5	2	160, 320
SØA13	62.3	67.8	2	160, 320
SØA14	110.2	97.3	1	320
SØA oppblom 8.8	204.0	409.6	2	640, 2560
SØA11	4068.6	4605.4	2	10240, 20480
SØA oppblom 8.8	2793.3	2970.4	4	2560, 5120, 10240, 20480
SV Alg 12.9	135.9	166.8	4	320, 640, 1280, 2560

Supporting information

Table S3. Information on minor impurities in the MC and NOD CRMs where accurate mass was used to confirm identities, and LC-HRMS was used to estimate their concentrations.

Minor impurity	*m/z* [M+H]$^+$	Δ ppm	Information values (µM)				
			CRM MCRR	CRM MCLR	CRM [Dha^7]MCLR	CRM MCLA	CRM NODR
[Asp^3]MC-RR*	512.78285	-0.958	0.03				
[Dha^7]MC-RR*	512.78286	-0.977	0.08				
[Glu(OMe)^6]MC-RR*	526.79847	-0.875	0.01				
[Asp^3]MC-LR	981.54128	-0.903		0.02			
[Glu(OMe)^6]MC-LR	1009.57268	-0.977		0.23			
[6(*Z*)-Adda^5,Dha^7]MC-LR	981.54142	-1.045			0.03		
[Asp^1]NOD-R	811.43524	-0.460					0.09
[Dha^5]NOD-R	811.43517	-0.373					0.07
[Glu(OMe)^4]NOD-R	839.46664	-0.565					0.02
([Glu(OMe)^6]MC-LA	924.50670	-0.054				0.02	
Conc CRM (µM)<br>% impurities (of total)			9.91<br>1.2 %	10.2<br>2.4 %	9.58<br>0.31 %	5.07<br>0.39 %	12.4<br>1.4 %

Supporting information

Table S4. Information on MC in-house RMs where accurate mass was used to confirm identities, and LC-HRMS was used to estimate the approximate purity.

Microcystin RM	*m/z* [M+H]$^+$	Δ ppm	approximate purity	information value (µM)
RM MC-YR	1045.5332	0.202	97%	0.51
RM MC WR	1068.5487	0.243	95%	0.60
RM MC-RY	1045.5324	0.278	92%	0.86
RM [Asp^3]MC-LR	981.5380	0.244	99%	0.73
RM [Asp^3]MC-RR	1024.5537	0.365	94%	0.55
RM MC-LF	986.5218	0.157	92%	0.62
RM MC-LW	1025.5320	0.219	96%	0.73
RM MC-LY	1002.5164	0.186	97%	0.61
RM [D-Leu^1]MC-LY	1044.5646	0.057	99%	7.44
RM MC-HtyR	1059.5494	0.147	99%	0.51
RM MC-HilR	1009.5691	0.257	95%	0.61
RM [D-Asp^3,(E)-Dhb^7]MC-RR	1024.5572	0.027	99%	0.31
[Glu(OMe)^6]MC-LA	924.5082	-0.054	99%	14.4
[$^{15}N_7$][D-Asp^3]MC-LA	917.4710	0.033	99%	8.22

References:

(1) Miles, C. O., et al., Microcystin profiles in European noble crayfish *Astacus astacus* and water in Lake Steinsfjorden, Norway. *Environ. Res.* **2024,** *242*, 117623. DOI: 10.1016/j.envres.2023.117623.
